# An edge-simplicity bias in the visual input to young infants

**DOI:** 10.1126/sciadv.adj8571

**Published:** 2024-05-10

**Authors:** Erin M. Anderson, T. Rowan Candy, Jason M. Gold, Linda B. Smith

**Affiliations:** ^1^ Psychological and Brain Sciences Department, Indiana University, Bloomington, IN 47405, USA.; ^2^ School of Optometry, Indiana University, Bloomington, IN 47405, USA.

## Abstract

The development of sparse edge coding in the mammalian visual cortex depends on early visual experience. In humans, there are multiple indicators that the statistics of early visual experiences has unique properties that may support these developments. However, there are no direct measures of the edge statistics of infant daily-life experience. Using head-mounted cameras to capture egocentric images of young infants and adults in the home, we found infant images to have distinct edge statistics relative to adults. For infants, scenes with sparse edge patterns—few edges and few orientations—dominate. The findings implicate biased early input at the scale of daily life that is likely specific to the early months after birth and provide insights into the quality, amount, and timing of the visual experiences during the foundational developmental period for human vision.

## Teaser

Sparse edge patterns dominate the visual world of young infants.

## INTRODUCTION

The first stage of mammalian visual cortex, V1, extracts localized edges, the initial representation for building meaningful percepts of objects and scenes. The tuning of the receptive fields of these cells is dependent on early visual experience [e.g., (*1*–*3*)]. In humans, a great deal is known about how early front-end abnormalities (cataracts, strabismus, anisometropia, and ptosis) disrupt human visual development by perturbing input to the visual cortex and how the timing of the abnormalities during early infancy has long-term consequences for vision (*4*–*8*). However, very little is known about the statistics of the everyday input that is the context for visual development. This is a major gap in a complete account of experience-dependent developmental process (*8*).

**Fig. 1.**
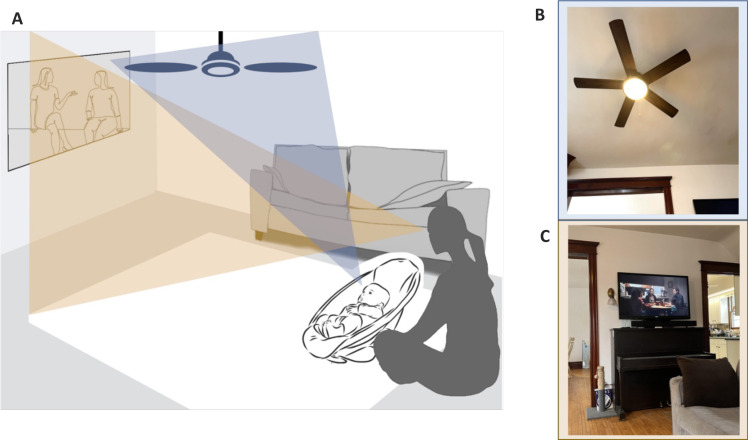
The egocentric view. (**A**) A hypothetical scene illustrating an adult and infant with different views despite close physical proximity. (**B** and **C**) Sampled scenes from the same room with different edge statistics.

By traditional assumptions, the massive scale of daily life presents pretty much the same visual statistics to all perceivers (*9*, *10*). Consistent with this assumption, seminal theoretical work has shown that sparse edge coding can emerge from the scene statistics found in large datasets of varied images (*10*–*12*). However, there are reasons to believe that the scene statistics experienced by young infants may not reflect a broader or an adult-like sampling of the visual world (*13*). At any moment, the images received at the eyes are spatially selective, depending on the location, posture, and behavior of the perceiver (Fig. 1A), properties that could yield unique input statistics for young infants.

In laboratory studies, young infants show specific and robust looking preferences (*5*, *14*–*16*). The stimuli in these studies consist of very simple arrays, typically line gratings or checkerboards. When shown two such images side-by-side, young infants systematically look for long durations at high contrast images with just a few extended edges. Given the extreme simplicity of the laboratory test images, it is unclear whether these in-lab looking preferences could bias the input at the scale of everyday experience. The world may rarely offer scenes with the preferred statistics, and even if such scenes were available, young infants may not be able to behaviorally select them. Young infants turn their heads to look with eyes and head pointed in the same direction (*17*–*20*), but their control of these movements is slow and variable (*20*, *21*). Moreover, young infants cannot move themselves from one location to another and thus must look at the world from the locations chosen by caregivers. These caregiver-chosen locations may not offer scenes with the preferred properties. However, if the visual world does offer such opportunities, and if young infants show the same looking preferences in everyday contexts that they do in laboratories, then the early input statistics could be unique to this period of development (Fig. 1, B and C).

To test this hypothesis, we analyzed a corpus of egocentric images collected by head cameras worn by 10 infants (3 to 13 weeks of age) as they went about their daily life. We chose this age range because it is a period characterized by robust looking preferences in the laboratory (*14*–*16*, *20*), rapid development of contrast sensitivity (*5*), and high vulnerability to the negative consequences of disrupted input (*6*–*8*). We also analyzed head-camera images collected by 10 adults (31 to 71 years of age) in the same community as the infant data collection. Most images from both age groups were captured inside their homes. Head cameras capture the head-centered field of view (FOV). Because unconstrained humans, including those as young as 3 weeks (*17*) as well as adults (*22*), rarely sustain gaze to the periphery of their FOV but instead look with eyes and heads pointed in the same direction (*17*–*19*, *22*), the scene statistics of a sufficiently large sample of at-home head-camera images provide a reasonable estimate of the scene statistics received by the eyes in that context (*23*). We analyzed 25,000 infant- and 25,000 adult-egocentric images downsampled from 70 hours of recorded head-camera video.

## RESULTS

**Fig. 2.**
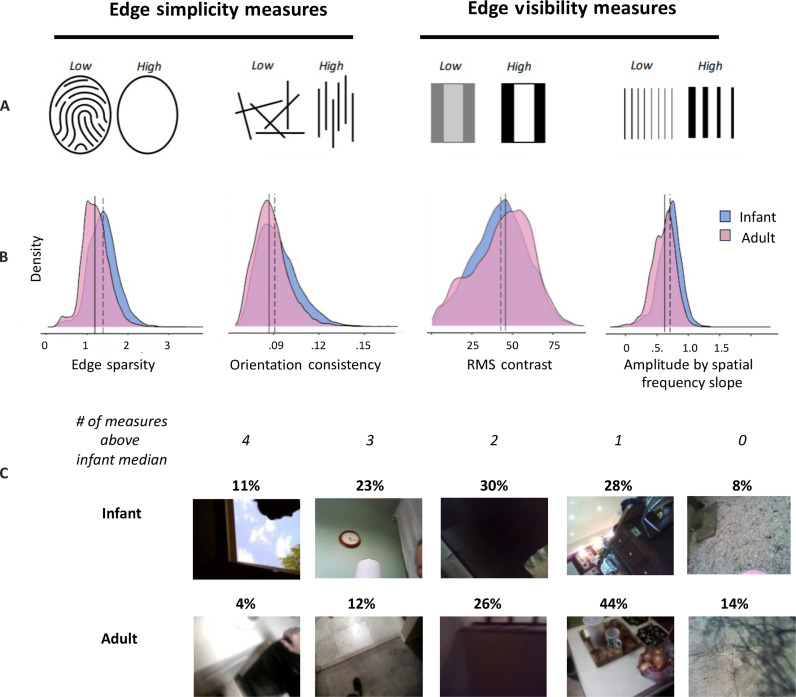
Edge measures. (**A**) Illustration of the four edge measures: sparsity, orientation consistency, RMS contrast, and amplitude by spatial frequency slope. (**B**) Density distribution of image values on the four measures. The solid vertical line indicates the median value of the adult images, and the dashed line is the infant median. (**C**) Percentage of images with “high” values, above the infant median values for both infants and adults, on 4, 3, 2, 1, and 0 of the four measures and example images with corresponding number of joint high values.

The visual arrays that elicit sustained looking by infants in the laboratory are characterized by very simple edge patterns and high contrast. Both components, the simplicity of edge patterns and contrast, predict infant visual attention (*14*–*16*). If infant looking in the home is similarly biased as that in controlled laboratory studies, then the infant egocentric images should differ from adults on these same two dimensions: the simplicity of the edge patterns and the contrast properties of the edges. Accordingly, we measured the images using two measures of edge simplicity and two measures of edge visibility (Fig. 2A). Simplicity depends on the number of edges and their patterning (*24*). To assess the edge simplicity, we measured each image with respect to (i) edge sparsity, the number of pixels belonging to an edge contour as measured by an edge detecting algorithm (*25*), and (ii) orientation consistency, the percent of edge pixels belonging to the dominant orientation in an image (*26*). Edge visibility depends on contrast; to assess this, we used two measures: (i) the amount of contrast of the whole image measured as the root mean square (RMS) contrast and (ii) the slope of the amplitude spectrum—the amount of log contrast as a function of log spatial frequency, an index of the proportion of contrast energy occurring at lower spatial frequencies.

### Edge simplicity and visibility

The frequency distributions of values on each of the four measures do not differ greatly between the infant and adult images (Fig. 2B). However, it is the joint properties of contrast and edge simplicity in a scene that determine infant preferential looking in the laboratory and the real-time neural response. We chose a first index of co-occurring properties that is simple, direct, and transparent as it requires no transformations of the raw data. We categorized each image as to whether it had a “high” or “low” value on each of the measures using the infant median on each measure as the threshold for high. We then counted how many (four, three, two, one, or zero) of the four measures were high. Infant and adult images differed (Fig. 2C) in the number of images with multiple high values across the four measures [χ^2^ (4, *N* = 50,000) = 2727.1, *P* < 0.001]. For infants, 36% of the infant images had values above the infant medians on at least three of the four measures, and most infant images (64%) fell above the infant medians on at least two of the four measures. In contrast, most adult images (57%) were characterized by just one or no measures with a value above the infant median (Fig. 2B). The fact that these co-occurrences of high values within an image on the four measures differ between infants and adults tells us that their images are distributed differently in the four-dimensional (4D) measurement space.

We created a theoretically motivated 2D visualization space to visualize these observed differences with respect to the simplicity of the edge patterns (edge sparsity and orientation consistency) and with respect to edge visibility (RMS contrast and amplitude by spatial frequency slope). To reduce the four measures to the two theoretically relevant dimensions, we performed two independent principal components analyses (PCAs). One reduced the two simplicity measures into a single edge-simplicity dimension (the first component of the PCA, which accounted for 60% of the total variance). The second PCA reduced RMS contrast and the slope of amplitude by spatial frequency to a visibility dimension (the first component that explained 62% of the total variance of the images). For each PCA, we used only the infant images, which are of central interest to this study. We then projected the two infant-based PCAs onto the adult images for comparison. Figure S1 shows the joint distribution of images on the visibility measures and on the two simplicity measures.

**Fig. 3.**
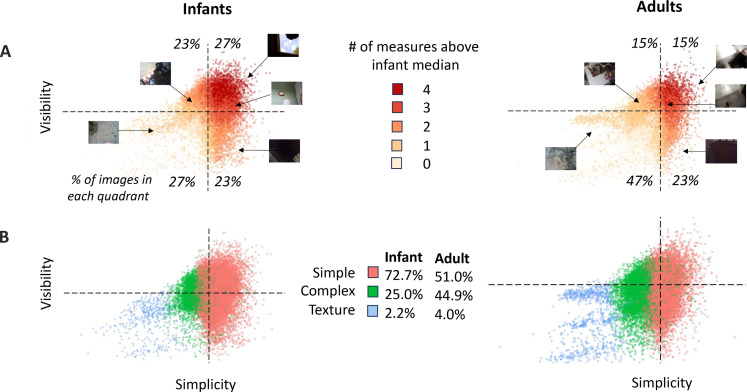
A visibility by simplicity visualization space. (**A**) Joint values of each infant and adult image, indicated by a single dot. The *x* axis is the simplicity dimension (principal component of edge sparsity and orientation consistency), and the *y* axis is the visibility dimension (principal component of RMS contrast and amplitude by spatial frequency slope). Dashed lines indicate the infant medians for visibility and simplicity. The percentage of images in quadrants defined by the PCA medians are given in the four corners of the space. Color shade indicates whether the image is above the infant median on 4, 3, 2, 1, or 0 measures (measures are edge sparsity, orientation consistency, RMS contrast, and amplitude by spatial frequency slope). Thumbnail images locate the example images from Fig. 2C in the simplicity by visibility space. (**B**) Distribution of infant and adult images in the simplicity by visibility space, with color indicating which of the three clusters (Simple, Complex, and Texture) the image was categorized as in the cluster analysis. The table gives the percent of images in each category for adults and infants.

Figure 3A shows the distribution of images with high values on four, three, two, one, and zero of the four measures (sparsity, orientation consistency, RMS contrast, and amplitude by spatial frequency slope) in the 2D visualization space. For both infants and adults, images that have more than two of the four measures above the infant medians fall on the high simplicity (right) side of the visualization space. Images with just one or zero of the four measures above their infant medians, the most frequent images for adults, rarely present simple edge patterns, and most of the images, by the standard of being above the infant median, are not high in visibility. The overall distribution of images in the four quadrants of the visualization space reflects these differences in the adult and infant images [χ^2^ (3, *N* = 50,000) = 2283, *P* < 001]. More infant images (27%) than adult images (15%) fell within the upper right quadrant of the visualization space, where images were marked by both high (infant) visibility and high (infant) simplicity [χ^2^ (1, *N* = 50,000) = 1089.1, *P* < 0.001].

Our second measure of the joint properties used bottom-up cluster analysis. Our purpose in the use of the cluster analysis was not to determine well-differentiated categories of images but rather as a basis for a data-determined definition of regions for the comparison of the joint properties of infant and adult images. Accordingly, we represented each image as a four-value vector using the untransformed measures for each image of edge sparsity, edge consistency, RMS contrast, and slope of amplitude by spatial frequency). We submitted the vector representations for each of the 25,000 infant images to a series of bottom-up *k*-means cluster analyses (*k* = 2, 3, 4; see fig. S2) (*27*). The three-cluster solution that provided the greatest differentiation between the infant images was highly consistent with nearly identical cluster nodes across 100 randomly generated starting seeds (SD < 0.0001 on all properties). Within the 2D visualization space, the three resulting clusters of infant images (Fig. 3B) present an almost linear ordering along the edge-simplicity axis (see fig. S1, A and B, for infant clusters with respect to the four contributing measures). In line with the ordering of the clusters on the simplicity axis of the visibility-by-simplicity visualization space (Fig. 3B), we labeled the clusters Simple (fewer edges and orientations), Complex (more edges and orientations), and Texture (many edges in many directions). Images in the Simple cluster made up 73% of the infant corpus. When we used the infant-derived clusters to characterize the adult data, the percent of images in the Simple cluster fell to 52% [χ^2^ (2, *N* = 50,000) = 2378, *P* < 0.00; see fig. S1, C and D, for the three clusters of adult images with respect to each of the four measures]. In sum, relative to adult egocentric images, the infant images are more frequently characterized by the co-occurrence of high edge visibility and high simplicity. Further, across the range of observed visibility values, there are many more infant egocentric images with simple edge patterns consisting of relatively few edges at few orientations.

### Image content

**Fig. 4.**
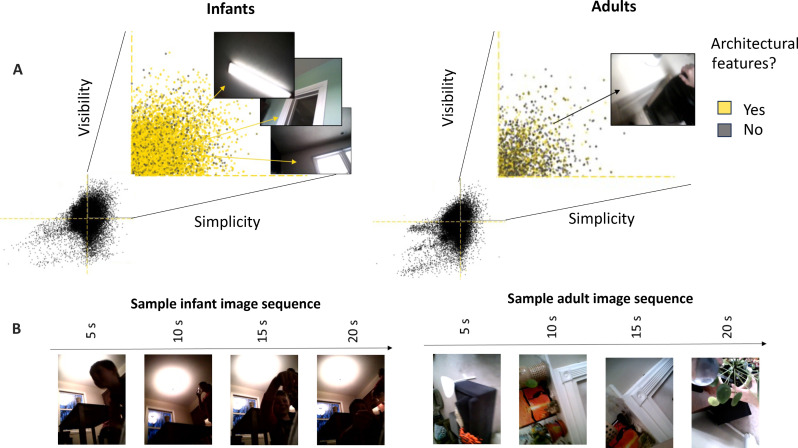
Content of scenes with high edge visibility and simplicity. (**A**) Infant and adult images that were (yellow) and were not (gray) judged to contain mostly architectural features that fell in the upper right quadrant of the visualization space. (**B**) Example image sequences from infant and adult visual streams. (Head-camera images were slightly cropped on the horizontal axis for displaying in figure.)

If one conceptualizes the present study as a preferential-looking experiment, the experimental stimuli were the scenes available in homes in a midwestern US town. From this perspective, our results constitute a “replication” in the uncontrolled environment of the home of infant preferences, relative to adults, to look at scenes with visible and simple edge patterns. What are these available simple edge scenes in homes? To answer this question, we asked human coders to label the content of a selected subset of the images, those occurring in the upper right quadrant of the 2D visualization space. Over 80% of the infant images in the selected region were labeled as containing windows, doors, walls, and lighting fixtures (Fig. 4A); only 30% of the adult images in this high-visibility high-simplicity quadrant were characterized as having this content. Within houses, large architectural edges and lighting fixtures create scenes of visible and simple edge patterns. Because infant preferential looking in the laboratory is measured by sustained looking to the preferred image, we also estimated how long images remained in the upper right quadrant of the visualization space by calculating the run length of successive images (sampled at 0.2 Hz for both the infant and adult images) within that quadrant. The mean run length was 3.8 images (19 s in duration) for infants and 2.9 images (14.5 s in duration) for adults. A multilevel model [Run length ~ group + (1 | participant)] confirmed that age group was a reliable predictor of the run length of consecutive images in the defined region of the visualization space, *F*_(1,19)_ = 5.53, *P* = 0.029, with an estimated ß = −0.78 (SE = 0.33) decrease from infant to adult consecutive content. In sum, adults, like infants, encounter scenes with simple visible edges, but these simple images do not remain in view for as long for adults (Fig. 4, A and B). However, in this context of daily-life activities, a series of self-similar egocentric scenes that endures over seconds characterizes both young infants’ and adults’ experiences. Because these images were captured by a head camera, the proximal cause of run length, for both infants and adults, is the sustained orientation of the head to the scene.

### Implications of biased sampling

There is no consensus on the precise ages at which critical periods for human visual development periods begin and end (*5*, *7*, *8*). However, measurable lifelong deficiencies in vision have been reported even when abnormalities were corrected by 4 months postnatal age (*5*, *7*, *8*), a fact that locates experiences in the early months after birth as critical to human visual development. Two translationally important questions raised by the present work concern whether and how much of the early input needs to consist of sparse edge patterns for typical development. The present data cannot answer these questions. However, our results provide an estimation of the amount of simple sparse edge patterns received by typically developing infants: 20 min per recorded hour of infant video contained images with at least three of the four measures at values above the infant medians, and 44 min per recorded hour consisted of images that fell in the Simple cluster. The aggregate number of normative awake hours (*28*) from birth to 3 months is about 720; by the present estimates, then, by the time they are 3 months of age, infants will have received 245 hours of exposure to images with at least three or four measures above the infant medians and 528 hours of images in the Simple cluster. These numbers provide a first estimate of the amount of simple and visible edge input that may be missing because of uncorrected abnormalities such as strabismus, anisometropia, and cataracts in the first 3 months after birth (*5*–*8*).

Some end-to-end machine learning models have been shown to perform better in downstream learning tasks when the model is pretrained with image datasets that have been submitted to low-pass filters [e.g., (*29*, *30*)]. The approach is characterized as mimicking early development. The underlying assumptions are, first, that the real-world input received at infant eyes is unbiased, variable, and complex; second, that the front-end immaturities responsible for the limited acuity act as a low-pass filter; and third, that this functional filter benefits the signal received at the cortical level (*29*, *30*). However, theorists of visual development (*5*,*14*) interpret young infants’ looking biases as reflecting their preference to look at scenes that can be clearly seen given their various front-end immaturities. This conceptualization places the bias at the selection of input and thus in the input received at the eye. To provide evidence on this issue, we asked to what degree a low-pass filter would alter the already biased input observed in the present study. We used contrast and color sensitivity data (*31*, *32*) to design a filter that approximated the sensitivities of an 8-week-old infant, which was the mean age of our infant sample. The low-pass filter resulted in 82% of the contrast in filtered images occurring at spatial frequencies below two cycles per degree and 87% below three cycles per degree. The filter further reduced luminance contrast by a factor of 0.4 and color saturation by a factor of 0.75.

**Fig. 5.**
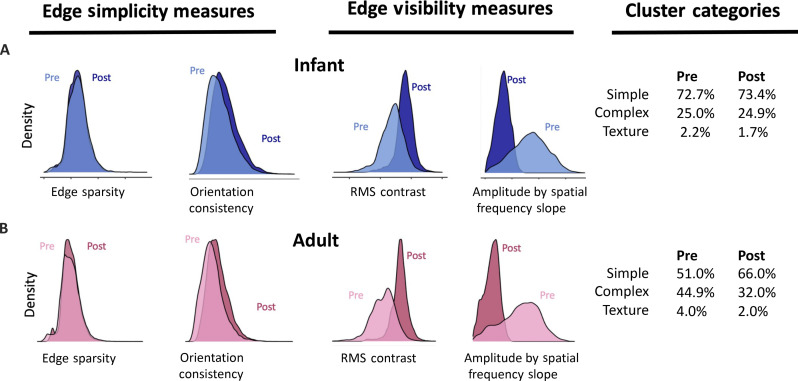
Effects of a low-pass filter. (**A**) Density distribution of infant image values on the four measures before and after filtering the images. (**B**) Density distribution of adult image values on the four measures before and after filtering the images. The tables show the percentage of images categorized according to the original cluster analysis based on infant prefiltered images.

For both the infant and adult images (Fig. 4, A and B), the major impact of a low-pass filter, which removes contrast at high spatial frequencies, is to increase the average contrast in the image and to reduce the amplitude by spatial frequency slope. The filter had minimal effects on edge sparsity and orientation consistency. We assessed the effects of the filter on the joint properties of individual images using the results of the original (pre-filter) cluster analysis of infant images to categorize all pre- and post-filter images. For the infant images, the percentages of images in the Simple, Complex, and Texture categories before and after filtering were statistically significant, [χ^2^ (2, *N* = 50,000) = 19.67, *P* < 0.001] but not meaningfully different as the percentage of images in each cluster changed by less than 1% (Fig. 5). For adults, the number of images in the Simple cluster increased by 15% after filtering [χ^2^ (2, *N* = 50,000) = 26.11, *P* < 0.001]. However, the number of post-filter adult images in the Simple cluster was still lower than that of pre-filter infant images in the Simple cluster [χ^2^ (2, *N* = 50,000) = 2158.3, *P* < 0.001]. Thus, the filtering hypothesis (*29*, *30*) is limited on two grounds. First, the output of a low-pass filter depends on the properties of the input received at the eye. For the daily-life experiences of infants captured in the present study, the input at the eye is sufficiently biased that a post-eye filter has minimal effects on the infant scene statistics. Second, the observed bias in the input to infants is perhaps not fully captured by a low-pass filter but may be better characterized as a bias for limited edge competition in a scene, that is, for scenes containing few edges at few orientations. Last, the finding of a strong bias at input to the eye has relevance for precortical visual development that also occurs in the early months after birth, including accommodation and convergence, which likely benefit from looking at targets that consist of few edges (*21*, *33*, *34*).

## DISCUSSION

A complete account of experience-dependent development requires a determination of how the developing system interacts with real-world environments (*8*). The present results provide a characterization of the edge statistics of daily-life visual input in the early months after birth, a period of rapid development within which disruptions in the expected input have lifelong consequences for vision (*5*–*8*). The results also provide a direct link from young infants’ looking preferences in the laboratory (*5*, *14*–*16*) to the statistics of daily experience.

Vision science has known since the work of Held and Hein (*1*) and Hirsch and Spinelli (*2*) that different timings and different classes of experiences matter for the development of different sensitivities (*6*). The bias for edge simplicity observed in laboratory-based preferential-looking studies is specific to the early months after birth; the edge-simplicity bias observed in infant egocentric images in the home is likely specific to this developmental period as well. The persistence of images with simple edge patterns, implicated by the long run lengths of images high in both edge visibility and simplicity, may also be a relevant property of daily-life experience. Slow change in the visual input has been demonstrated to yield more rapid learning in infants (*35*, *36*). By the principles of slow feature analysis (*37*–*39*), slow change in inputs that consist principally of sparse edge patterns would be expected to be optimal for a system learning to extract edges.

The present results concern the egocentric scene statistics of infants who live in man-made dwellings. Advances in vision science have emerged from the study of the statistics of natural (outdoor) scenes (*9*, *40*). It has been demonstrated that the mature visual system is well adapted to efficiently process images matching the biases in natural scene statistics (*9*, *40*). The larger theoretical idea behind the study of nature scenes (*40*) is that the visual statistics of the natural physical world imposed strong evolutionary constraints on mammalian (and thus human) vision. The images analyzed as natural scenes in these studies are not the egocentric experiences of active organisms in the wild but are photographs typically framed and taken by mature adults and thus potentially reflecting the biases of the mature visual system taking the photographs. Although a direct comparison of the statistics of infant egocentric images in carpentered homes and nature photographs has not been done, it is highly likely that the statistics of infant egocentric images collected in homes differ substantially from those of nature photographs. However, laboratory studies that tightly control the possible scenes to which infants can look have shown that by 4 months of age, infants exhibit at least some of the sensitivity biases predicted by natural scene statistics (*41*). Evolutionary constraints often play out through cascades of developmental change (*42*–*44*). A complete explanation of experience-dependent visual development will require an integrative account of natural scenes statistics with developmental changes in egocentric scene statistics and developmental changes in visual sensitivities.

An additional question raised by the present findings concerns the more proximal causes underlying the observed simplicity bias in the daily-life visual statistics of young infants. The correspondence between the simple edge patterns in the home and the scenes that young infants choose to look at in the laboratory strongly suggests that the bias originates in the infants and that it arises because infants visually orient to patterns they can clearly see (*14*). Although functional acuity in early infancy is the likely key factor underlying the observed statistics in the present study, a direct measure of individual infants’ visual sensitivities in controlled studies and looking behavior in the uncontrolled context of daily life is needed. How preferences for sparse edge scenes play out in infants’ real time behavior is also not yet determined. Although young infants can move their heads to orient to preferred views and shift posture to some degree, their movements are slow and variable (*17*–*20*). Further, young infants are not mobile, so the captured head-camera images depend on the locations in which they are placed by mature partners. One possibility is that infant gaze is first directed to visible and simple edge patterns and then the infants slowly orient their head in the direction of the gaze point. Alternatively, and consistent with the long in-view run lengths of scenes with visible simple edge patterns, the infant may just continue looking at simple patterns for long durations when they happen to be in view without any fussing that might cause a mature caregiver to alter the view.

Looking preferences depend not only on the intrinsic biases of the perceiver and their behavior but also on the visual scenes available to the perceiver. The biases apparent in infants’ looking preferences in the laboratory are limited with respect to larger conclusions about the statistics of daily-life experience because of the extremely simple and highly controlled images needed to measure infant preferences with precision. The findings from the present study are limited because they provide evidence from one daily-life context. If the infant bias for sparse and simple edge patterns is general and potent, infants may well be able to find scenes that meet their preferences in a variety of human contexts. The question of how much the visual ecology of young infants varies across contexts is critical for determining the quality and quantity of simple edge patterns in early input and their role in the developing visual system.

In conclusion, the results indicate an early visual world dominated by simple edge patterns: few edges at few orientations. This fact opens an agenda for empirical research on the role of simple edge scenes in post-birth visual development and its underlying causes.

## MATERIALS AND METHODS

### Apparatus

The head cameras were selected for ease of use by parents in the home without the presence of experimenters present and with care for the safety of the infant wearing the camera. The cameras (Looxcie 2 head cameras) were lightweight (2 oz) and did not heat up, and the recorded video was stored directly on the camera. The head cameras (*18*, *23*) had a resolution of 720 × 1280 pixels and recorded at 30 frames/s. The diagonal FOV of the camera was 75°, vertical FOV was 42°, and horizontal FOV was 69° with 2″ to infinity depth of focus. The battery life of each camera (without recharging) was one to three continuous hours.

### Human subjects

Ten infants (5 males, 3 to 13 weeks of age) and 10 adults (3 males, 31 to 71 years of age) contributed data. The infants were recruited from a parent opt-in participant database. The adults were recruited from employees at the university. All recruitment, consenting, procedural, analysis, and storage of data procedures were reviewed and approved by the Institutional Review Board of Indiana University, protocol number 1505862312.

### Data collection

At a preliminary visit, parents were trained to place the cameras on the infant head so that it did not wobble and was directly above the infant nose. Adult participants were given similar training in placing the cameras on themselves. All recordings took place during daily-life activities without experimenters being present. Participating families were given two to four fully charged cameras and asked to record up to 6 hours of video while the infant or adult was wearing the camera and awake. Video stored on the camera during data collection was transferred to secure computers for storage and processing.

### Image sampling

The infants contributed 41.5 hours, and adults contributed 30.75 hours. To sample the same quantity of images from adults and infants (25,000 from each age group) for analyses of image properties, we sampled the infant videos at one frame every 5 s and the adult images at one frame every 4 s. For the temporal analyses of run lengths of simple visible edge scenes, we sampled both infant and adult images at one frame every 5 s.

### Image property measures

All measures and the resulting datasets are archived in Dataverse (https://dataverse.harvard.edu/dataset.xhtml?persistentId=doi:10.7910/DVN/PNWYPZ) and in an OSF repository (https://osf.io/5pkuz/?view_only=1b02cbbf3ab945cdb7f3fae94f93a728). For each of the following image property measures, the images were first converted to grayscale.

### Amplitude by spatial frequency

To determine that contrast was measured rather than luminance, the image was converted by subtracting the average luminance value of the image window from the individual values, where the image window was a cosine window with a radius of 0.4 times the image height (in this case, 480 pixels). The image was converted to Fourier space, and amplitude was measured at 240 spatial frequencies ranging from 0 to 5.8 cycles per degree.

### Orientation consistency

Using Simoncelli and Freeman’s (*26*) steerable pyramids design, we measured the amplitude at 16 orientations and four spatial frequency bands, summing amplitude across these bands. We then identified the dominant orientation, where the greatest cumulative amplitude in the image occurred. The percent of amplitude at the dominant orientation for each image became the measure of orientation consistency. RMS contrast was the SD of pixel luminance over the entire (grayscale) image.

### Edge sparsity

Each image was passed through MATLAB’s built-in edge() function, using the Canny (*25*) edge detector method, a lower threshold of 0.10, and an SD (σ) of 0.25. These settings were chosen because human judges determined that they best captured the major contours of shapes within these images, without picking up fine texture. The number of pixels representing part of an edge was summed and then reverse-coded to provide a measure of sparsity rather than complexity.

### The 2D visualization space

On the basis of the properties of the scenes to which infants preferentially look in laboratory studies, we defined a priori two dimensions of edge properties, visibility (contrast properties) and simplicity (number and orientation of edges). We independently performed two PCAs to combine RMS contrast and amplitude by spatial frequency into the visibility dimension and number of edges and edge orientation consistency into the simplicity dimension. For the PCA on amplitude by spatial frequency and contrast, the first component, our defined visibility dimension, explained 62% of the total variance of the images on the two contributing measures. For the PCA on edge sparsity and orientation consistency, the first component, which defined our simplicity dimension, explained 60% of the total variance.

### Cluster analysis

We used a *k*-means cluster analysis (*27*), initially comparing two-, three-, and four-cluster solutions. The three-cluster solution provided the greatest differentiation between images and was highly consistent, with nearly identical cluster nodes across 100 randomly generated starting seeds (SD < 0.0001 on all properties). The initial cluster analysis was performed on the prefiltered infant images. A *k*-nearest neighbor classification was used to then categorize the adult images and the post-filtered images from both age groups, using the class package in R (*45*).

### Coding image content

Images of interest (those high in edge simplicity and edge visibility) were coded by humans through Amazon Mechanical Turk. Coders were asked to determine whether each image contained any of the following elements: “Window,” “Door / Doorway,” “Ceiling,” “Light,” “Floor,” or “Corner (where walls meet),” to select the relevant elements or to select “None of the above” if the image did not contain any of these elements. We chose these elements for human coding because they seemed extremely common by our own judgements. Subjects were shown positive and negative examples of such images before they began their selections. Each coder judged 25 images at a time, and each image was shown to two independent coders. In total, 969 coders completed the task and had an agreement level of 90%. For our analyses, images were only considered to contain one or more elements if both coders agreed.

### Image filter

We ran a Gaussian filter with SD (σ) of 2 over each image, reducing the proportion of energy at spatial frequencies above two cycles per degree from *M* = 32% to *M* = 18%. We further scaled the luminance contrast down by a factor of 0.4 using the following approach

$$\left(L_{i}-X_{L}\right)*\text{CF}+X_{L}$$

where *L*_i_ is the luminance of an individual pixel, *X*_L_ is the mean luminance across the image, and CF is the contrast factor (0.4).

This reduced the overall contrast from *M* = 41.61 (16.28) in the original images to *M* = 16.45 (SD = 6.42) after filtering.

Last, we scaled the color intensity down slightly (75% of the original), in line with the evidence that 8-week-old infants are generally able to differentiate color when luminance is controlled for, although photoreceptor development is ongoing (*14*). The script for the filter is available in Dataverse (https://dataverse.harvard.edu/dataset.xhtml?persistentId=doi:10.7910/DVN/PNWYPZ) and the following OSF repository (https://osf.io/5pkuz/?view_only=1b02cbbf3ab945cdb7f3fae94f93a728).
