# Supplementary material for: An edge-simplicity bias in the visual input to young infants
Source: Sci Adv. 2024 May 10;10(19):eadj8571. doi: 10.1126/sciadv.adj8571 (PMC11086614; doi:10.1126/sciadv.adj8571)
Supplement: Supplementary file 1 — Figs. S1 and S2 [file sciadv.adj8571_sm.pdf]

Science Advances
AAAS

Supplementary Materials for

An edge-simplicity bias in the visual input to young infants

Erin M. Anderson *et al.*

Corresponding author: Linda B. Smith, smith4@iu.edu

Sci. Adv. **10**, eadj8571 (2024)
DOI: 10.1126/sciadv.adj8571

This PDF file includes:

Figs. S1 and S2

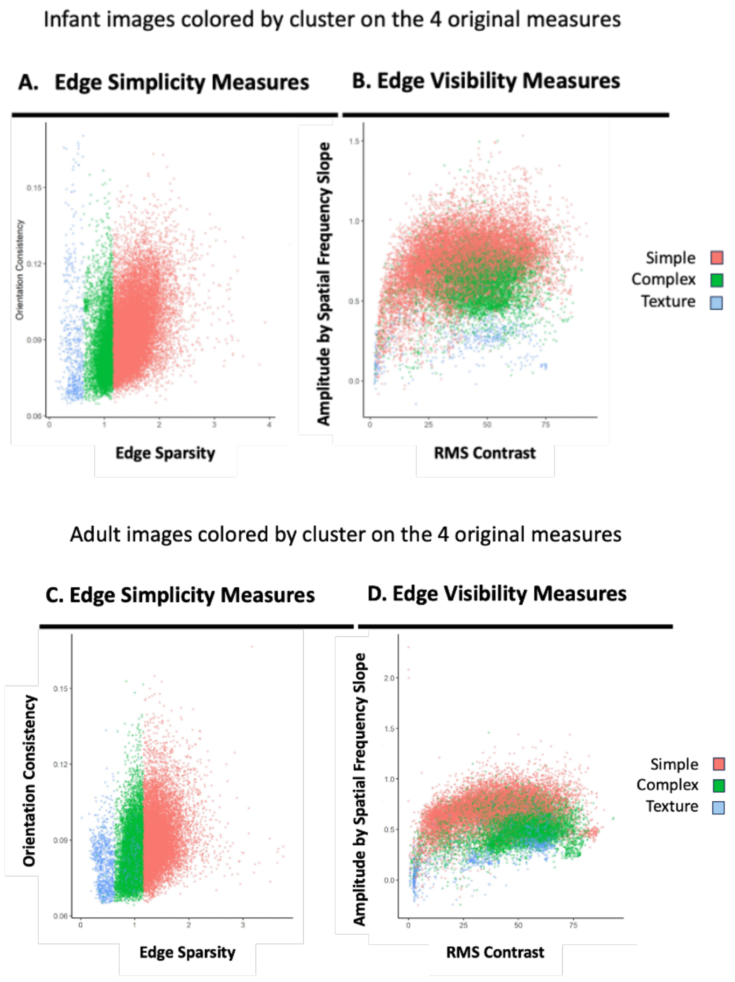

Fig. S1.

Distribution of images on the four measures. (A) The joint distribution of infant images on the two Simplicity measures of Orientation Consistency and Edge Sparsity. (B) The joint distribution of the infant images on the two Visibility measures of Amplitude by Spatial Frequency Slope and RMS contrast. (C) The joint distribution of adult images on the two Simplicity measures of Orientation Consistency and Edge Sparsity. (D) The joint distribution of the adult images on the two Visibility measures of Amplitude by Spatial Frequency Slope and RMS contrast. The color of the dots indicates membership in the three clusters (Simple, Complex, and Texture) determined by the cluster analysis.

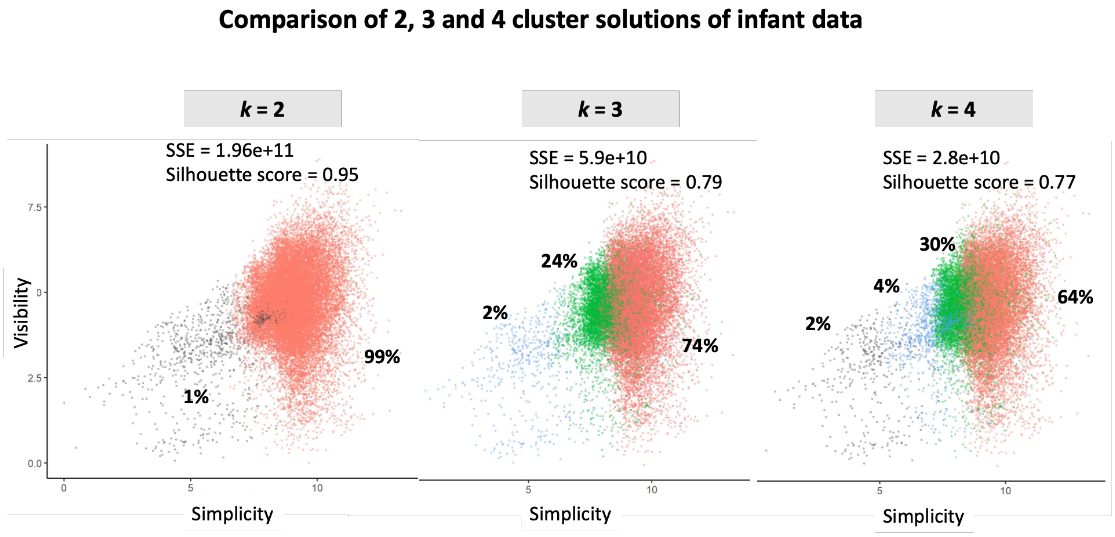

Fig. S2.

Cluster solutions for k=2, 3, 4. Each infant image was represented a four-value vector using the untransformed measures for each image of edge sparsity, edge consistency, RMS contrast, and slope of amplitude by spatial frequency. These vector representations were submitted to a series of bottom-up k-means cluster analyses with the observed solutions at k =2, 3, 4 shown in the Visibility by Simplicity visual space. SSE indicates the level of cohesion within clusters, with smaller numbers corresponding with greater cohesion. The silhouette score indicates both the level of intra-cluster similarity and inter-cluster distance, with a higher score suggesting more distinct clusters. The 3-cluster solution best balances the two metrics. Additionally, the 3-cluster solution has more descriptive power than the two-cluster solution (in which 99% of the images are in one group), while the 4-cluster solution does not show much qualitative difference from the 3-cluster solution.
